# Roles of Biomarkers in Chronic Kidney Disease and Related Conditions

**DOI:** 10.3390/jcm15155920

**Published:** 2026-07-29

**Authors:** Takayasu Ohtake, Shuzo Kobayashi

**Affiliations:** 1 Regenerative Medicine, Shonan Kamakura General Hospital, Kamakura 247-8533, Japan; 2 Shonan Research Institute of Innovative Medicine (sRIIM), Kamakura 251-8555, Japan; 3 Kidney Disease & Transplant Center, Shonan Kamakura General Hospital, Kamakura 247-8533, Japan

Chronic kidney disease (CKD) is a major clinical problem with increasing incidence and mortality worldwide. Therefore, efforts should be made to prevent onset and the progression of CKD. The Special Issue, “Chronic Kidney Disease: Clinical Challenges and Management,” in the *Journal of Clinical Medicine* was initiated to address the growing need for a deeper understanding of CKD and to stimulate multidisciplinary discussion among clinicians, researchers, and allied health care professionals. Seven articles were published in this Special Issue. Among these, four reported on the usefulness of novel biomarkers for CKD or CKD-related conditions.

Kobayashi et al. [1] reported that asymmetric dimethylarginine (ADMA), an endogenous inhibitor of nitric-oxide synthase that is usually considered as a marker of endothelial dysfunction and atherosclerosis, was significantly and independently associated with the progression of coronary artery calcification in predialysis patients with CKD. In addition, ADMA was significantly associated with the progression of kidney dysfunction, as reflected by a decline in the estimated glomerular filtration rate (eGFR). Therefore, ADMA may be considered an important novel biomarker for both cardiovascular and kidney diseases, and intervention with ADMA may improve cardiorenal outcomes in patients with CKD.

Rusu et al. [2] reported a relationship between high-sensitivity C-reactive protein (hs-CRP), an important biomarker of both inflammation and survival, and adipose tissue biomarkers in patients undergoing hemodialysis. High adipose mass was positively associated with hs-CRP levels. These researchers demonstrated a complex relationship among inflammation, adipose tissue biomarkers, and nutritional status in patients undergoing hemodialysis.

Kato et al. [3] reported that elevated urinary liver-type fatty-acid-binding protein (L-FABP) levels predicted renal function decline in diabetic and hypertensive patients with preserved eGFR. As urinary L-FABP is thought to reflect tubulointerstitial damage associated with renal microcirculatory impairment, it has potential utility as a urinary biomarker for the early diagnosis and prognosis of CKD.

Enoki et al. [4] provided a novel heatmap to predict the probability of reaching an eGFR < 30 mL/min/1.73 m^2^ by age 80 years of age. By applying single eGFR data and age alone, this heatmap could predict the lifetime risk of kidney failure. The authors proposed that multiple eGFR measurements are not necessary and that age-adjusted risk stratification based on a single eGFR measurement will be clinically useful for guiding surveillance intensity and earlier intervention strategies.

Biomarkers in the field of CKD have not yet been fully established as diagnostic tools. Nevertheless, they play increasing vital roles in elucidating disease pathophysiology [5,6], stratifying risk [7], guiding therapeutic selection [8], evaluating therapeutic efficacy [9], predicting prognosis [10], and facilitating precision nephrology [11]. Further evolution in CKD biomarkers is expected, particularly in the fields of disease progression prediction and precision medicine.

## References

[1] Kobayashi S., Ohtake T., Mochida Y., Ishioka K., Oka M., Maesato K., Moriya H., Hidaka S. (2025). Asymmetric dimethylarginine (ADMA) as a novel risk factor for progression of coronary artery calcification in patients with chronic kidney disease. J. Clin. Med..

[2] Rusu C.C., Kacso I., Moldovan D., Potra A., Tirinescu D., Ticala M., Masyyennikov Y., Urs A., Bondor C.I. (2025). Exploring the associations between inflammatory biomarkers, survival, and cardiovascular events in hemodialysis patients and the interrelationship with nutritional parameters—The experience of a single Transylvanian Dialysis Center. J. Clin. Med..

[3] Kato Y., Sugaya T. (2026). Verification of the utility of urinary L-FABP as a predictor of impaired renal function based on its relationship with changes in renal function. J. Clin. Med..

[4] Enoki R., Miyazaki M., Imai E., Tanaka T., Okamoto K. (2026). Predicting lifetime risk of kidney failure using age and a single eGFR measurement. J. Clin. Med..

[5] Li X., Raisinghani N., Gallinat A., Santos-Gallego C.G., Zhang S., La Salvia S., Yoon S., Yavuz H., Pham A., Shao A. (2026). Circulating extracellular vesicles in the pathogenesis of heart failure in patients with chronic kidney disease. Circulation.

[6] Jung C.Y., Yoo T.H. (2022). Pathophysiologic mechanisms and potential biomarkers in diabetic kidney disease. Diabetes Metab. J..

[7] Zeng X., Liu D., Su L., Wang M., Yang G., Liang M., Peng Z. (2026). The creatinine-cystatin C ratio as a prognostic risk-stratification biomarker in chronic kidney disease. Front. Nutr..

[8] Ergunay T., Brossa A., Bussolati B. (2026). Diagnostic and therapeutic roles of extracellular vesicles in chronic kidney disease: A systematic review. J. Extracell. Vesicles.

[9] Si S., Liu H., Xu L., Zhan S. (2024). Identification of novel therapeutic targets for chronic kidney disease and kidney function by integrating multi-omics proteome with transcriptome. Genome Med..

[10] Swaminathan S.M., Rao I.R., Shenoy S.V., Prabhu A.R., Mohan P.B., Rangaswamy D., Bhojaraja M.V., Nagri S.K., Nagaraju S.P. (2023). Novel biomarkers for prognosticating diabetic kidney progression. Int. Urol. Nephrol..

[11] Stefaneu E., Tountas C., Inannidis E., Kole C. (2024). Biomarkers in cardiorenal syndrome, a potential use in precision medicine. J. Nephrol..

